# Crystal structure of bis­[1,3,4,5-tetra­methyl-1*H*-imidazole-2(3*H*)-thione-κ*S*]chlorido­copper(I)

**DOI:** 10.1107/S1600536814024404

**Published:** 2014-11-15

**Authors:** Ulrich Flörke, Aziza Ahmida, Hans Egold, Gerald Henkel

**Affiliations:** a Department Chemie, Fakultät für Naturwissenschaften, Universität Paderborn, Warburgerstrasse 100, D-33098 Paderborn, Germany

**Keywords:** crystal structure, trigonal coordination, copper, imidazole

## Abstract

The mol­ecular structure of the title compound, [CuCl(C_7_H_12_N_2_S)_2_], shows a slightly distorted trigonal–planar coordination geometry of the Cu atom. The Cu—Cl bond measures 2.2287 (9) Å, and the two Cu—S bonds are significantly different from each other, with values of 2.2270 (10) and 2.2662 (10) Å. Also, the S—Cu—Cl angles differ, with values of 113.80 (4) and 124.42 (4)°, while the S—Cu—S angle is 121.51 (4)°. The two imidazole rings are almost parallel, making a dihedral angle of 2.1 (2)°. In the crystal, the shortest C—H⋯Cl interactions stabilize a three-dimensional network with molecules linked into centrosymmetric dimers that are stacked along the *b*-axis direction.

## Related literature   

For structures of related Cu complexes, see: Devillanova *et al.* (1980 ▶); Kimani *et al.* (2011 ▶). For background to effective anti-oxidants, see: Bhabak *et al.* (2010 ▶); Yamashita & Yamashita (2010 ▶). 
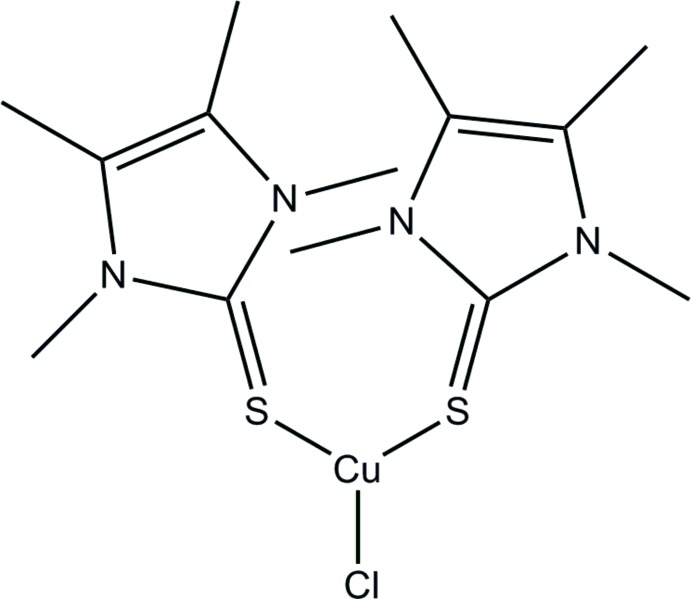


## Experimental   

### Crystal data  

[CuCl(C_7_H_12_N_2_S)_2_]
*M* *_r_* = 411.48
Monoclinic, 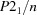

*a* = 9.4738 (14) Å
*b* = 13.662 (2) Å
*c* = 14.119 (2) Å
β = 98.314 (3)°
*V* = 1808.2 (5) Å^3^
*Z* = 4
Mo *K*α radiation
μ = 1.59 mm^−1^
*T* = 120 K
0.25 × 0.20 × 0.11 mm

### Data collection  

Bruker SMART CCD area-detector diffractometer
Absorption correction: multi-scan (*SADABS*; Bruker, 2002 ▶) *T*_min_ = 0.692, *T*_max_ = 0.845
17386 measured reflections
4304 independent reflections
2584 reflections with *I* > 2σ(*I*)
*R*_int_ = 0.099

### Refinement  

*R*[*F* ^2^ > 2σ(*F* ^2^)] = 0.047
*wR*(*F* ^2^) = 0.091
*S* = 0.85
4304 reflections
207 parameters
H-atom parameters constrained
Δρ_max_ = 0.52 e Å^−3^
Δρ_min_ = −0.58 e Å^−3^

### 

Data collection: *SMART* (Bruker, 2002 ▶); cell refinement: *SAINT* (Bruker, 2002 ▶); data reduction: *SAINT*; program(s) used to solve structure: *SHELXTL* (Sheldrick, 2008 ▶); program(s) used to refine structure: *SHELXTL*; molecular graphics: *SHELXTL*; software used to prepare material for publication: *SHELXTL* and local programs.

## Supplementary Material

Crystal structure: contains datablock(s) I, global. DOI: 10.1107/S1600536814024404/zq2228sup1.cif

Structure factors: contains datablock(s) I. DOI: 10.1107/S1600536814024404/zq2228Isup2.hkl

**Fig. 1.**
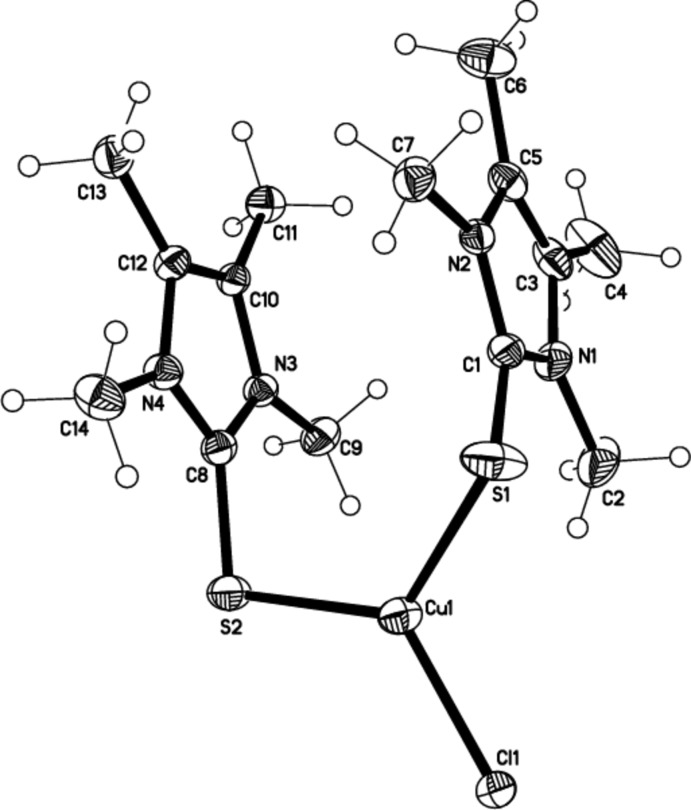
Molecular structure of the title compound with anisotropic displacement parameters drawn at the 50% probability level.

. DOI: 10.1107/S1600536814024404/zq2228fig1.tif
Mol­ecular structure of the title compound with anisotropic displacement parameters drawn at the 50% probability level.

CCDC reference: 1032971

Additional supporting information:  crystallographic information; 3D view; checkCIF report

## Figures and Tables

**Table 1:** Hydrogen-bond geometry (, )

*D*H*A*	*D*H	H*A*	*D* *A*	*D*H*A*
C4H4*B*Cl1^i^	0.98	2.75	3.717(4)	170
C11H11*A*Cl1^ii^	0.98	2.76	3.721(3)	165
C14H14*B*Cl1^iii^	0.98	2.80	3.782(4)	176

Symmetry codes: (i) 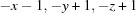; (ii) 
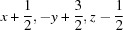
; (iii) 
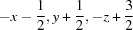
.

## supplementary crystallographic information

### S1. Comment

We are interested in the chemistry of *N*,*N*-dimethylimidazole-thione derivatives due to their ability to act as effective antioxidants (Bhabak *et al.*, 2010; Yamashita *et al.*, 2010). Here we report the synthesis of a copper(I) chloride complex with 1,3,4,5-tetra-methylimidazole-2-thione ligands.

The title compound shows the same *trans* configuration as the bis-*N*,*N*'-dimethylimidazole-thione-Cu(I) compound (Kimani *et al.*, 2011) or bis-*N*,*N*'-dimethylimidazolidine-thione-CuCl (Devillanova *et al.*, 1980) whereas the *cis* configuration is also known for bis-*N*,*N*'-dimethylimidazole-thione-CuX (*X* = Cl, Br, I) (Kimani *et al.*, 2011). In contrast to all the reported complexes in the title compound both Cu and Cl atoms lie on general positions and the two Cu—S bond lengths differ strongly with Cu–S1 2.2662 (10) and Cu–S2 2.2270 (10) Å. Also the S—Cu—Cl angles differ with 113.80 (4)° and 124.41 (4)°, while the S—Cu—S angle is 121.51 (4)°.

The intramolecular hydrogen bonds between the chlorine atom and hydrogen atoms of the methyl group amount to 4.838 (H2b—Cl) and 4.911 Å(H9a—Cl).

### S2. Experimental

To a solution of 1,3,4,5-tetra-methylimidazoline-2-thione (0.390 mg, 2.75 mmol) in acetonitrile (50 ml) CuCl_2_ (0.168 mg, 1.25 mmol) was added and the mixture was stirred at room temperature for 24 h. Afterwards the solvent was removed under vacuum. White crystals were obtained from diffusion of diethyl ether into acetonitrile.

### S3. Refinement

Hydrogen atoms were clearly identified in difference syntheses, refined at idealized positions riding on the carbon atoms with isotropic displacement parameters *U*_iso_(H) = 1.5*U*_eq_(–CH_3_) and C–H = 0.98 Å. All CH_3_ hydrogen atoms were allowed to rotate but not to tip.

### Crystal data

**Table:**

[CuCl(C_7_H_12_N_2_S)_2_]	*F*(000) = 856
*M**_r_* = 411.48	*D*_x_ = 1.512 Mg m^−^^3^
Monoclinic, *P*2_1_/*n*	Mo *K*α radiation, λ = 0.71073 Å
*a* = 9.4738 (14) Å	Cell parameters from 2398 reflections
*b* = 13.662 (2) Å	θ = 2.6–23.8°
*c* = 14.119 (2) Å	µ = 1.59 mm^−^^1^
β = 98.314 (3)°	*T* = 120 K
*V* = 1808.2 (5) Å^3^	Prism, blue
*Z* = 4	0.25 × 0.20 × 0.11 mm

### Data collection

**Table:**

Bruker SMART CCD area-detector diffractometer	4304 independent reflections
Radiation source: sealed tube	2584 reflections with *I* > 2σ(*I*)
Graphite monochromator	*R*_int_ = 0.099
φ and ω scans	θ_max_ = 27.9°, θ_min_ = 2.1°
Absorption correction: multi-scan (*SADABS*; Bruker, 2002)	*h* = −12→12
*T*_min_ = 0.692, *T*_max_ = 0.845	*k* = −17→16
17386 measured reflections	*l* = −18→18

### Refinement

**Table:**

Refinement on *F*^2^	Primary atom site location: structure-invariant direct methods
Least-squares matrix: full	Secondary atom site location: difference Fourier map
*R*[*F*^2^ > 2σ(*F*^2^)] = 0.047	Hydrogen site location: difference Fourier map
*wR*(*F*^2^) = 0.091	H-atom parameters constrained
*S* = 0.85	*w* = 1/[σ^2^(*F*_o_^2^) + (0.0349*P*)^2^] where *P* = (*F*_o_^2^ + 2*F*_c_^2^)/3
4304 reflections	(Δ/σ)_max_ = 0.001
207 parameters	Δρ_max_ = 0.52 e Å^−^^3^
0 restraints	Δρ_min_ = −0.58 e Å^−^^3^

### Special details

**Table:**

Geometry. All e.s.d.'s (except the e.s.d. in the dihedral angle between two l.s. planes) are estimated using the full covariance matrix. The cell e.s.d.'s are taken into account individually in the estimation of e.s.d.'s in distances, angles and torsion angles; correlations between e.s.d.'s in cell parameters are only used when they are defined by crystal symmetry. An approximate (isotropic) treatment of cell e.s.d.'s is used for estimating e.s.d.'s involving l.s. planes.
Refinement. Refinement of *F*^2^ against ALL reflections. The weighted *R*-factor *wR* and goodness of fit *S* are based on *F*^2^, conventional *R*-factors *R* are based on *F*, with *F* set to zero for negative *F*^2^. The threshold expression of *F*^2^ > σ(*F*^2^) is used only for calculating *R*-factors(gt) *etc*. and is not relevant to the choice of reflections for refinement. *R*-factors based on *F*^2^ are statistically about twice as large as those based on *F*, and *R*- factors based on ALL data will be even larger.

### Fractional atomic coordinates and isotropic or equivalent isotropic displacement parameters (Å^2^)

**Table:**

	*x*	*y*	*z*	*U*_iso_*/*U*_eq_	
Cu1	−0.42892 (4)	0.63369 (3)	0.67946 (3)	0.02856 (13)	
Cl1	−0.50281 (8)	0.51059 (6)	0.76351 (6)	0.0275 (2)	
S1	−0.18839 (9)	0.64545 (9)	0.69146 (7)	0.0453 (3)	
S2	−0.57219 (9)	0.74573 (7)	0.60170 (6)	0.0308 (2)	
N1	−0.1745 (3)	0.5835 (2)	0.5081 (2)	0.0312 (7)	
N2	−0.0533 (3)	0.71469 (19)	0.54686 (19)	0.0215 (6)	
N3	−0.4380 (3)	0.77570 (19)	0.44451 (19)	0.0224 (6)	
N4	−0.3860 (3)	0.88515 (19)	0.55378 (19)	0.0246 (7)	
C1	−0.1411 (3)	0.6492 (2)	0.5796 (2)	0.0264 (8)	
C2	−0.2621 (4)	0.4972 (3)	0.5142 (3)	0.0497 (12)	
H2A	−0.2016	0.4387	0.5193	0.075*	
H2B	−0.3333	0.4926	0.4567	0.075*	
H2C	−0.3106	0.5021	0.5709	0.075*	
C3	−0.1069 (3)	0.6088 (3)	0.4301 (3)	0.0325 (9)	
C4	−0.1248 (4)	0.5507 (3)	0.3408 (3)	0.0578 (13)	
H4A	−0.0819	0.5860	0.2916	0.087*	
H4B	−0.2266	0.5406	0.3187	0.087*	
H4C	−0.0776	0.4872	0.3529	0.087*	
C5	−0.0309 (3)	0.6901 (3)	0.4548 (2)	0.0273 (8)	
C6	0.0632 (4)	0.7501 (3)	0.4023 (3)	0.0421 (10)	
H6A	0.0681	0.7205	0.3396	0.063*	
H6B	0.1591	0.7528	0.4391	0.063*	
H6C	0.0244	0.8165	0.3935	0.063*	
C7	0.0058 (3)	0.8007 (2)	0.5986 (3)	0.0301 (8)	
H7A	−0.0400	0.8098	0.6559	0.045*	
H7B	−0.0113	0.8584	0.5573	0.045*	
H7C	0.1087	0.7918	0.6174	0.045*	
C8	−0.4618 (3)	0.8033 (2)	0.5325 (2)	0.0238 (8)	
C9	−0.5003 (4)	0.6902 (2)	0.3929 (3)	0.0320 (9)	
H9A	−0.5329	0.6440	0.4382	0.048*	
H9B	−0.4286	0.6586	0.3597	0.048*	
H9C	−0.5816	0.7106	0.3460	0.048*	
C10	−0.3462 (3)	0.8419 (2)	0.4094 (2)	0.0229 (8)	
C11	−0.3028 (3)	0.8309 (3)	0.3134 (2)	0.0285 (8)	
H11A	−0.2349	0.8828	0.3034	0.043*	
H11B	−0.3870	0.8358	0.2644	0.043*	
H11C	−0.2576	0.7669	0.3088	0.043*	
C12	−0.3132 (3)	0.9106 (2)	0.4776 (2)	0.0248 (8)	
C13	−0.2251 (3)	1.0006 (2)	0.4780 (3)	0.0314 (8)	
H13A	−0.1750	1.0005	0.4220	0.047*	
H13B	−0.1553	1.0023	0.5365	0.047*	
H13C	−0.2868	1.0583	0.4759	0.047*	
C14	−0.3773 (4)	0.9387 (3)	0.6430 (3)	0.0350 (9)	
H14A	−0.4383	0.9969	0.6336	0.052*	
H14B	−0.2784	0.9587	0.6638	0.052*	
H14C	−0.4095	0.8967	0.6919	0.052*	

### Atomic displacement parameters (Å^2^)

**Table:**

	*U*^11^	*U*^22^	*U*^33^	*U*^12^	*U*^13^	*U*^23^
Cu1	0.0284 (2)	0.0310 (3)	0.0266 (2)	−0.0035 (2)	0.00511 (17)	0.0040 (2)
Cl1	0.0260 (4)	0.0266 (5)	0.0307 (5)	−0.0004 (4)	0.0067 (4)	0.0052 (4)
S1	0.0254 (5)	0.0805 (9)	0.0295 (5)	−0.0085 (5)	0.0023 (4)	0.0173 (6)
S2	0.0249 (4)	0.0367 (6)	0.0314 (5)	−0.0010 (4)	0.0059 (4)	0.0092 (4)
N1	0.0220 (14)	0.0205 (16)	0.049 (2)	−0.0061 (13)	−0.0017 (14)	0.0012 (15)
N2	0.0190 (13)	0.0205 (15)	0.0247 (16)	−0.0006 (11)	0.0024 (11)	−0.0018 (12)
N3	0.0215 (14)	0.0216 (16)	0.0239 (16)	−0.0023 (12)	0.0021 (11)	0.0001 (12)
N4	0.0237 (14)	0.0258 (17)	0.0246 (16)	−0.0023 (12)	0.0045 (12)	−0.0019 (13)
C1	0.0163 (15)	0.028 (2)	0.034 (2)	−0.0002 (14)	0.0005 (14)	0.0082 (17)
C2	0.031 (2)	0.027 (2)	0.087 (4)	−0.0087 (18)	−0.008 (2)	0.009 (2)
C3	0.0194 (17)	0.038 (2)	0.039 (2)	0.0031 (16)	0.0017 (16)	−0.0098 (18)
C4	0.035 (2)	0.073 (3)	0.063 (3)	0.001 (2)	0.000 (2)	−0.041 (3)
C5	0.0221 (17)	0.035 (2)	0.025 (2)	0.0030 (15)	0.0012 (14)	0.0000 (16)
C6	0.037 (2)	0.062 (3)	0.028 (2)	−0.009 (2)	0.0079 (17)	0.005 (2)
C7	0.0293 (18)	0.026 (2)	0.034 (2)	−0.0014 (16)	0.0013 (16)	−0.0028 (17)
C8	0.0200 (16)	0.0241 (19)	0.027 (2)	0.0005 (14)	0.0007 (14)	0.0051 (15)
C9	0.034 (2)	0.026 (2)	0.036 (2)	−0.0061 (16)	0.0028 (16)	−0.0009 (17)
C10	0.0211 (16)	0.023 (2)	0.0247 (19)	0.0016 (14)	0.0031 (14)	0.0060 (15)
C11	0.0272 (18)	0.033 (2)	0.024 (2)	−0.0006 (15)	0.0008 (15)	0.0046 (16)
C12	0.0262 (17)	0.0231 (19)	0.026 (2)	−0.0001 (15)	0.0047 (15)	0.0043 (16)
C13	0.0283 (18)	0.028 (2)	0.037 (2)	−0.0005 (16)	0.0021 (16)	−0.0017 (17)
C14	0.033 (2)	0.042 (2)	0.030 (2)	−0.0038 (17)	0.0078 (17)	−0.0098 (18)

### Geometric parameters (Å, º)

**Table:**

Cu1—S2	2.2270 (10)	C4—H4C	0.9800
Cu1—Cl1	2.2287 (9)	C5—C6	1.486 (5)
Cu1—S1	2.2662 (10)	C6—H6A	0.9800
S1—C1	1.704 (4)	C6—H6B	0.9800
S2—C8	1.721 (3)	C6—H6C	0.9800
N1—C1	1.354 (4)	C7—H7A	0.9800
N1—C3	1.395 (4)	C7—H7B	0.9800
N1—C2	1.451 (4)	C7—H7C	0.9800
N2—C1	1.348 (4)	C9—H9A	0.9800
N2—C5	1.388 (4)	C9—H9B	0.9800
N2—C7	1.452 (4)	C9—H9C	0.9800
N3—C8	1.349 (4)	C10—C12	1.349 (5)
N3—C10	1.395 (4)	C10—C11	1.480 (4)
N3—C9	1.456 (4)	C11—H11A	0.9800
N4—C8	1.339 (4)	C11—H11B	0.9800
N4—C12	1.402 (4)	C11—H11C	0.9800
N4—C14	1.449 (4)	C12—C13	1.486 (4)
C2—H2A	0.9800	C13—H13A	0.9800
C2—H2B	0.9800	C13—H13B	0.9800
C2—H2C	0.9800	C13—H13C	0.9800
C3—C5	1.342 (5)	C14—H14A	0.9800
C3—C4	1.479 (5)	C14—H14B	0.9800
C4—H4A	0.9800	C14—H14C	0.9800
C4—H4B	0.9800		
			
S2—Cu1—Cl1	124.42 (4)	H6A—C6—H6C	109.5
S2—Cu1—S1	121.51 (4)	H6B—C6—H6C	109.5
Cl1—Cu1—S1	113.80 (4)	N2—C7—H7A	109.5
C1—S1—Cu1	109.23 (11)	N2—C7—H7B	109.5
C8—S2—Cu1	102.52 (11)	H7A—C7—H7B	109.5
C1—N1—C3	109.8 (3)	N2—C7—H7C	109.5
C1—N1—C2	124.6 (3)	H7A—C7—H7C	109.5
C3—N1—C2	125.5 (3)	H7B—C7—H7C	109.5
C1—N2—C5	110.2 (3)	N4—C8—N3	106.5 (3)
C1—N2—C7	125.3 (3)	N4—C8—S2	127.2 (3)
C5—N2—C7	124.5 (3)	N3—C8—S2	126.3 (3)
C8—N3—C10	110.1 (3)	N3—C9—H9A	109.5
C8—N3—C9	125.1 (3)	N3—C9—H9B	109.5
C10—N3—C9	124.8 (3)	H9A—C9—H9B	109.5
C8—N4—C12	110.0 (3)	N3—C9—H9C	109.5
C8—N4—C14	125.3 (3)	H9A—C9—H9C	109.5
C12—N4—C14	124.7 (3)	H9B—C9—H9C	109.5
N2—C1—N1	105.9 (3)	C12—C10—N3	106.8 (3)
N2—C1—S1	126.6 (3)	C12—C10—C11	131.2 (3)
N1—C1—S1	127.4 (3)	N3—C10—C11	122.0 (3)
N1—C2—H2A	109.5	C10—C11—H11A	109.5
N1—C2—H2B	109.5	C10—C11—H11B	109.5
H2A—C2—H2B	109.5	H11A—C11—H11B	109.5
N1—C2—H2C	109.5	C10—C11—H11C	109.5
H2A—C2—H2C	109.5	H11A—C11—H11C	109.5
H2B—C2—H2C	109.5	H11B—C11—H11C	109.5
C5—C3—N1	106.9 (3)	C10—C12—N4	106.7 (3)
C5—C3—C4	131.0 (4)	C10—C12—C13	130.6 (3)
N1—C3—C4	122.1 (3)	N4—C12—C13	122.7 (3)
C3—C4—H4A	109.5	C12—C13—H13A	109.5
C3—C4—H4B	109.5	C12—C13—H13B	109.5
H4A—C4—H4B	109.5	H13A—C13—H13B	109.5
C3—C4—H4C	109.5	C12—C13—H13C	109.5
H4A—C4—H4C	109.5	H13A—C13—H13C	109.5
H4B—C4—H4C	109.5	H13B—C13—H13C	109.5
C3—C5—N2	107.1 (3)	N4—C14—H14A	109.5
C3—C5—C6	131.7 (3)	N4—C14—H14B	109.5
N2—C5—C6	121.2 (3)	H14A—C14—H14B	109.5
C5—C6—H6A	109.5	N4—C14—H14C	109.5
C5—C6—H6B	109.5	H14A—C14—H14C	109.5
H6A—C6—H6B	109.5	H14B—C14—H14C	109.5
C5—C6—H6C	109.5		
			
S2—Cu1—S1—C1	−58.01 (14)	C1—N2—C5—C6	−179.5 (3)
Cl1—Cu1—S1—C1	127.70 (13)	C7—N2—C5—C6	2.5 (5)
Cl1—Cu1—S2—C8	−168.08 (12)	C12—N4—C8—N3	0.4 (3)
S1—Cu1—S2—C8	18.26 (13)	C14—N4—C8—N3	−178.3 (3)
C5—N2—C1—N1	−0.3 (3)	C12—N4—C8—S2	−178.0 (2)
C7—N2—C1—N1	177.8 (3)	C14—N4—C8—S2	3.3 (5)
C5—N2—C1—S1	175.2 (2)	C10—N3—C8—N4	−0.4 (3)
C7—N2—C1—S1	−6.7 (5)	C9—N3—C8—N4	−179.6 (3)
C3—N1—C1—N2	−0.1 (4)	C10—N3—C8—S2	177.9 (2)
C2—N1—C1—N2	177.1 (3)	C9—N3—C8—S2	−1.2 (5)
C3—N1—C1—S1	−175.6 (2)	Cu1—S2—C8—N4	−94.7 (3)
C2—N1—C1—S1	1.7 (5)	Cu1—S2—C8—N3	87.3 (3)
Cu1—S1—C1—N2	130.1 (3)	C8—N3—C10—C12	0.3 (4)
Cu1—S1—C1—N1	−55.3 (3)	C9—N3—C10—C12	179.5 (3)
C1—N1—C3—C5	0.5 (4)	C8—N3—C10—C11	−179.0 (3)
C2—N1—C3—C5	−176.7 (3)	C9—N3—C10—C11	0.1 (5)
C1—N1—C3—C4	−179.4 (3)	N3—C10—C12—N4	0.0 (3)
C2—N1—C3—C4	3.4 (5)	C11—C10—C12—N4	179.2 (3)
N1—C3—C5—N2	−0.6 (4)	N3—C10—C12—C13	−177.2 (3)
C4—C3—C5—N2	179.2 (4)	C11—C10—C12—C13	2.1 (6)
N1—C3—C5—C6	179.4 (3)	C8—N4—C12—C10	−0.2 (4)
C4—C3—C5—C6	−0.7 (7)	C14—N4—C12—C10	178.5 (3)
C1—N2—C5—C3	0.6 (4)	C8—N4—C12—C13	177.2 (3)
C7—N2—C5—C3	−177.5 (3)	C14—N4—C12—C13	−4.1 (5)

### Hydrogen-bond geometry (Å, º)

**Table:**

*D*—H···*A*	*D*—H	H···*A*	*D*···*A*	*D*—H···*A*
C2—H2*C*···S1	0.98	2.74	3.217 (4)	110
C4—H4*B*···Cl1^i^	0.98	2.75	3.717 (4)	170
C7—H7*A*···S1	0.98	2.73	3.209 (3)	110
C9—H9*A*···S2	0.98	2.77	3.211 (4)	108
C11—H11*A*···Cl1^ii^	0.98	2.76	3.721 (3)	165
C14—H14*B*···Cl1^iii^	0.98	2.80	3.782 (4)	176
C14—H14*C*···S2	0.98	2.77	3.223 (4)	109

Symmetry codes: (i) −*x*−1, −*y*+1, −*z*+1; (ii) *x*+1/2, −*y*+3/2, *z*−1/2; (iii) −*x*−1/2, *y*+1/2, −*z*+3/2.
