# Supplementary figures and images for: Crystal structure of bis­[1,3,4,5-tetra­methyl-1H-imidazole-2(3H)-thione-κS]chlorido­copper(I)
Source: Acta Crystallogr Sect E Struct Rep Online. 2014 Nov 15;70(Pt 12):m397–8. doi: 10.1107/S1600536814024404 (PMC4257435; doi:10.1107/S1600536814024404)

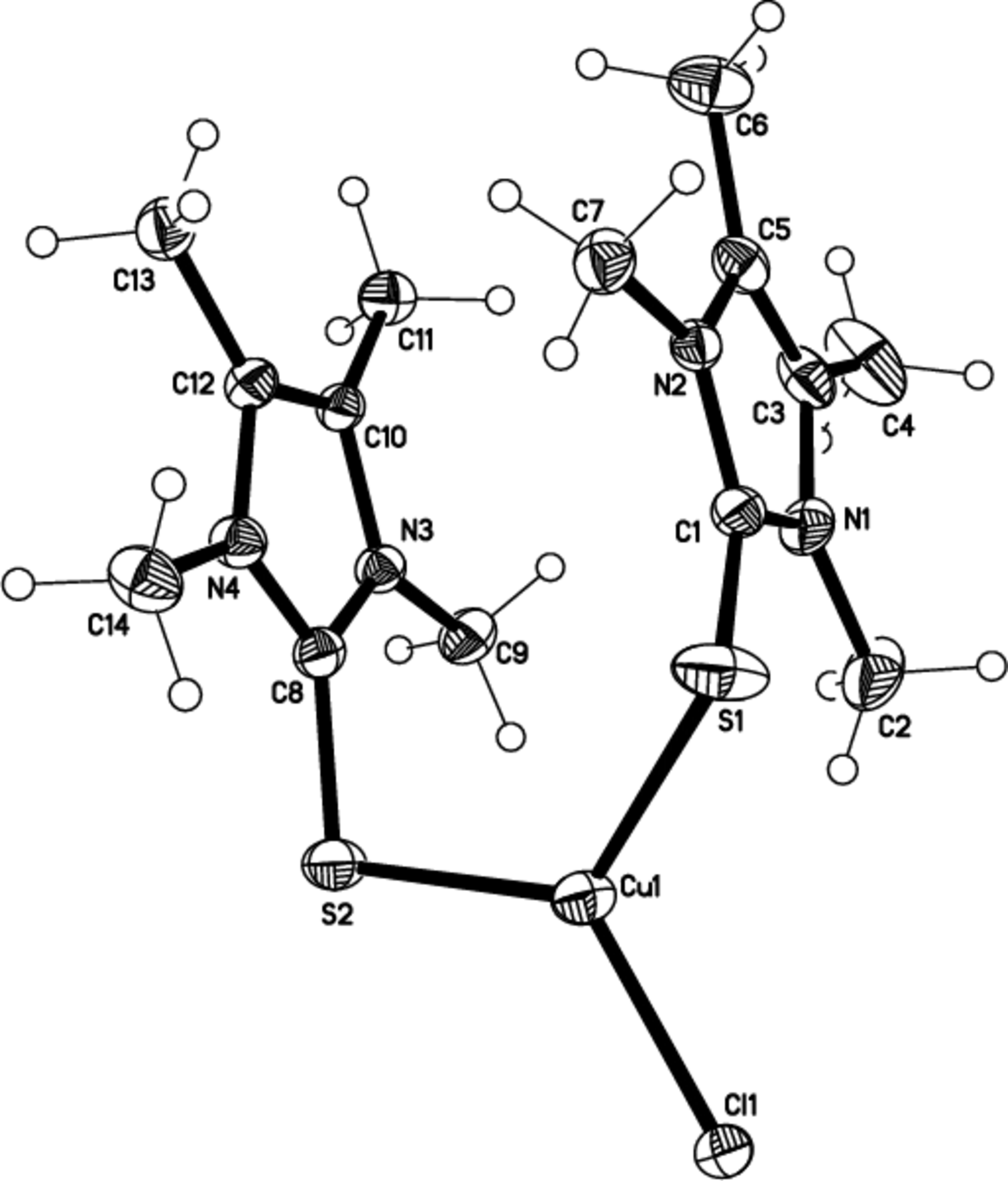

Supplement: Supplementary file 3 [file e-70-0m397-fig1.tif]
